# Periosteal wrapping of the hamstring tendon autograft improves graft healing and prevents tunnel widening after anterior cruciate ligament anatomic reconstruction

**DOI:** 10.1007/s00402-024-05356-9

**Published:** 2024-05-15

**Authors:** Ahmed Helal, Osama El-Gebaly, Hany Hamed, Ali M. Omran, ElSayed ELForse

**Affiliations:** 1 Department of Orthopaedics, Tanta Faculty of Medicine, Tanta University, Tanta, El-Gharbia Governorate Egypt; 2 Department of Orthopaedics, Faculty of Medicine, Kafr El-Shaikh University, Kafr El Sheikh, Egypt

**Keywords:** ACL reconstruction, Knee ligament reconstruction, Periosteum augmentation

## Abstract

**Introduction:**

The periosteum is a readily available tissue at the hamstring harvest site that could be utilized to enhance graft healing and prevent tunnel widening without additional cost or morbidity. This study aimed to compare graft healing using magnetic resonance imaging (MRI) and functional clinical outcome scores in a matched cohort of patients who underwent anterior cruciate ligament (ACL) reconstruction with hamstring autografts with or without periosteal augmentation.

**Material and methods:**

Forty-eight patients who underwent ACL reconstruction (ACLR) were prospectively enrolled: 25 with standard ACLR (ST-ACLR) and 23 with periosteal augmented grafts (PA-ACLR). The same surgical techniques, fixation methods, and postoperative protocol were used in both groups. Signal-to-noise quotient (SNQ), graft healing at the bone-graft interface, graft signal according to the Howell scale, and femoral tunnel widening were evaluated using MRI after 1 year of follow-up. International knee documentation score (IKDC), Lysholm, Tegner activity scale, and visual analog scale for pain were used for functional evaluation at a minimum of 2 years postoperative.

**Results:**

The mean SNQ of the proximal part of the graft was 9.6 ± 9.2 and 2.9 ± 3.3 for the ST-ACLR and PA-ACLR groups, respectively (*P* = 0.005). The mean femoral tunnel widening was 30.3% ± 18.3 and 2.3% ± 9.9 for the ST-ACLR, PA-ACLR groups, respectively (*P* < 0.001). Complete graft tunnel healing was observed in 65% and 28% of cases in the PA-ACLR and ST-ACLR groups, respectively. Both groups showed marked improvements in functional scores, with no statistically significant differences.

**Conclusion:**

Periosteal wrapping of hamstring tendon autografts is associated with better graft healing and maturation and lower incidence of femoral tunnel widening based on MRI analysis 1 year after ACL reconstruction. However, patient-reported outcomes and measured laxity were similar between the two groups at 2 years follow up.

**Trial registration:**

Trail registration number: PACTR202308594339018, date of registration: 1/5/2023, retrospectively registered at the Pan African Clinical Trial Registry (pactr.samrc.ac.za) database.

## Introduction

Hamstring tendon autografts are widely used for anterior cruciate ligament (ACL) reconstruction [1]. However, slower graft-tunnel incorporation, graft maturation, and tunnel widening remain some of the biggest concerns with hamstring autografts compared with BTB autografts [2–4]. Several studies have investigated different methods for enhancing graft-tunnel healing to increase osteointegration, including the use of bone morphogenic proteins, platelet-derived growth factors, transforming growth factor-b1 (TGF-b1), bone substitutes, and periosteum autografts [5–7].

The periosteum is a natural, readily available, osteogenic tissue that consists of two histologically different layers: the outer fibrous layer and the inner cambium layer that comprise mesenchymal progenitor cells or undifferentiated precursor cells capable of differentiating into either osteoblasts or chondrocytes depending on the culture environment [7]. The clinical outcomes of ACL reconstruction with periosteum-augmented hamstring tendon grafts have been previously reported, with encouraging results regarding functional and radiological parameters [8, 9]. Most of these studies used transtibial femoral drilling and two small free periosteal flaps sutured to the graft at the femoral and tibial tunnel apertures [7–9].

The present study aimed to compare the radiological findings using MRI scan obtained at 12 months postoperative and functional clinical outcome scores in a matched cohort of patients who underwent ACL reconstruction with hamstring autografts, either with or without periosteal augmentation with a minimum clinical follow-up period of 24 months. It was hypothesised that patients who underwent ACL reconstruction with periosteal augmentation would have better graft-tunnel healing, maturation, and less tunnel widening, with subsequently better functional outcomes at a 2-year follow up.

## Material and methods

This prospective cohort study was conducted in accordance with the principles of the Declaration of Helsinki. This study was approved by the local ethical committee (ethical approval code: 36264PR207/1/17). In addition, the study received approval from our institutional review board and all patients provided informed consent for participation. Patients who underwent primary single-bundle ACL reconstruction between February 2017 and January 2020 were prospectively enrolled. All procedures were performed by two surgeons (A. H. and S. F.) at the same institution using an autologous hamstring graft.

The inclusion criteria were (1) Skeletally mature male patients aged between 18 and 50 years, (2) Isolated ACL injury confirmed clinically (positive Lachman, pivot shift, and anterior drawer tests) and radiologically on MRI, (3) Healthy contralateral knee, (4) Absence of previous injuries in the affected knee, (5) Intact or repaired menisci at the time of surgery, (6) Low grade pivot shift (Grade 0–2) detected on examination under anesthesia, (7) Posterior tibial slope (PTS) measured on lateral plain radiograph < 12, (8) ACL graft diameter more than 8 mm, and (9) Patients with less than 3 months of interval between injury and surgery. Further intraoperative criteria included preserved tibial remnants (Crain type I–III) after graft implantation covering at least 25% of the intraarticular graft length. The following exclusion criteria were applied: (1) Posterior cruciate ligament (PCL), lateral and medial collateral ligament injuries higher than grade 2; (2) Cartilage damage or osteoarthritic changes (Outerbridge classification > 2), and (3) Meniscal deficiencies or tears that underwent partial meniscectomy.

In total, 59 patients were enrolled in the study. Patients were assigned consecutively into two groups in a 1:1 balanced allocation: a periosteal augmentation ACL reconstruction (PA-ACLR) and standard ACL reconstruction group (ST-ACLR) groups. Four patients were lost during the follow-up period, four patients did not undergo follow-up MRI examination, and three patients (1.7%) had to undergo a second surgery for varied reasons (graft rupture, cyclops resection, and meniscal retear) prior to the final clinical assessment and, therefore, 11 patients were excluded (7 patients from PA-ACLR group and 4 patients from ST-ACLR group). Ultimately, 48 patients were included in the analysis: 23 patients in the (PA-ACLR) group and 25 patients in the (ST-ACLR) group. The patient demographics are presented in Table 1.

**Table 1:** Patients' demographics and differences between both groups

	ST-ACLR (*n* = 25)	PA-ACLR (*n* = 23)	*P* value
Age at time of surgery (years)	24.5 ± 7.6 years	26.7 ± 8.1 years	0.992
Body mass index kg/m^2^	23.6 ± 2.7 kg/m^2^	25.2 ± 3.7 kg/m^2^	0.843
Time from injury to surgery (mean)	27 days	29 days	0.791
Pre-injury Tegner score (median)	6	7	0.038^*^
Drilled femoral tunnel diameter (Mean)	8.5 mm	9 mm	0.047^*^
Meniscal injuries			
Medial meniscus	*N* = 7 (30%)	*N* = 4 (9%)	0.711
Lateral meniscus	*N* = 6 (25%)	*N* = 6 (26%)	
Follow up period in months, mean (range)	27 (24–30)	30 (24–36)	0.283
Time from surgery to MRI (months)	12.5 ± 7.7	14 ± 5.0	0.671

^*^Statstically significant

### Surgical technique

**Fig. 1:**
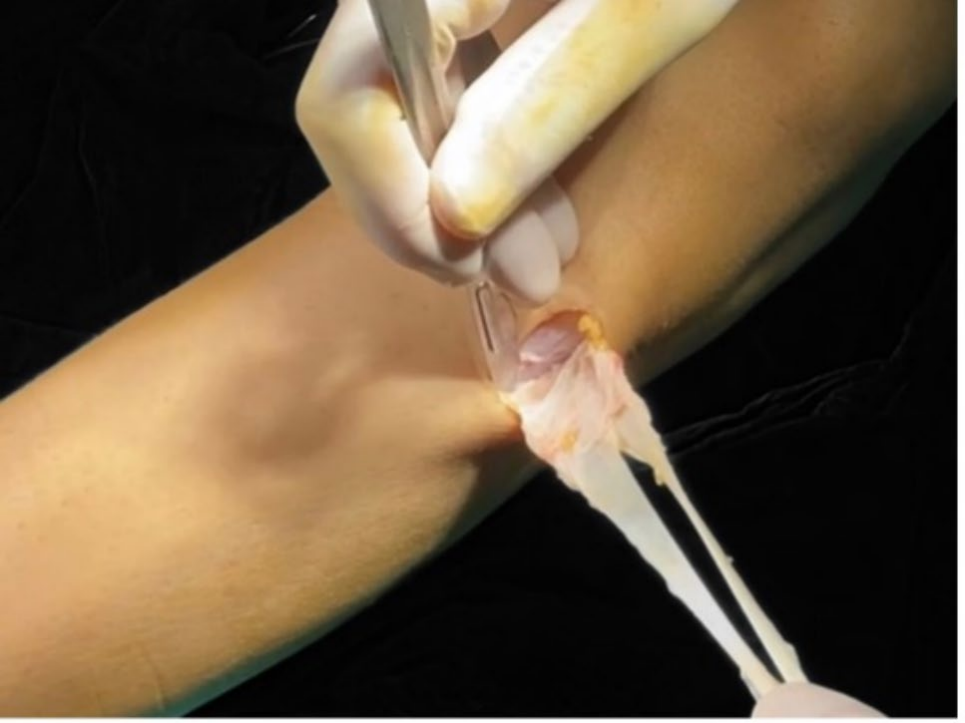
(Right knee) the gracilis and semitendinosus tendons are harvested together with the periosteum attached to the distal end, which is sharply dissected from the bone using a scalpel

After receiving regional anesthesia, the patients were placed in a supine position on the operating table with the leg flexed to 90º and kept in position with foot and lateral support. Both the gracilis and semitendinosus tendons were harvested using an open stripper, and by the aid of a sharp scalpel and a periosteal elevator the distal ends of the hamstring grafts were harvested and freed from the tibial attachment together with an attached periosteal flap measuring approximately 1.5 × 3 cm (Fig. 1).

**Fig. 2:**
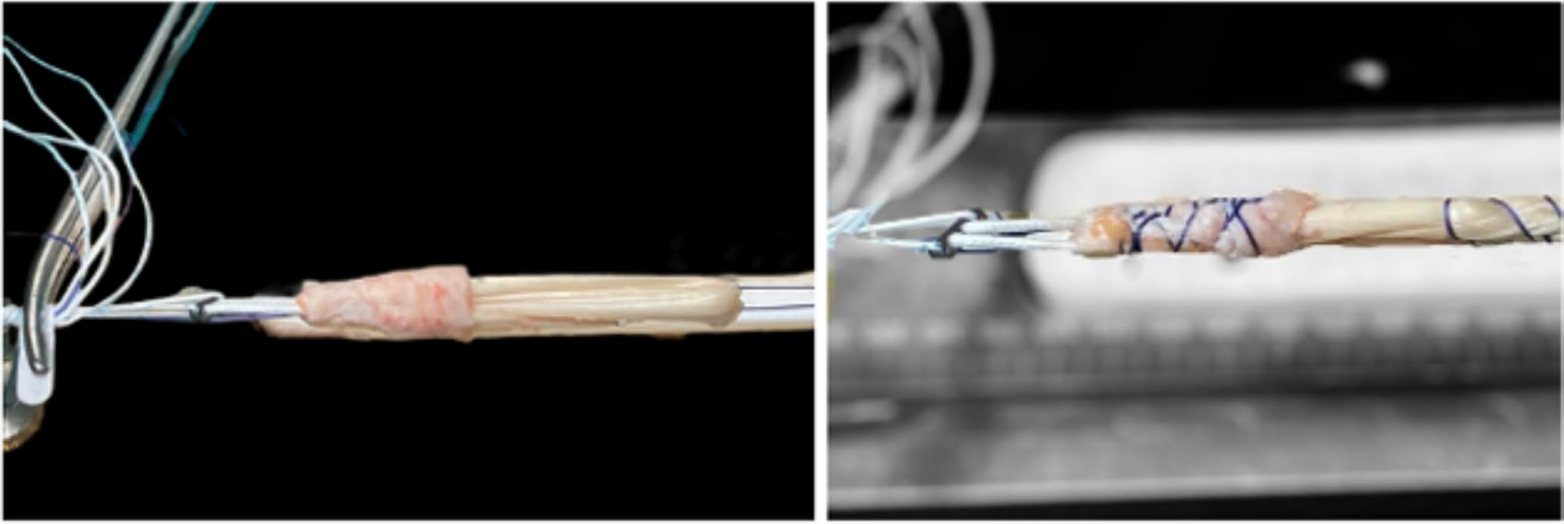
The periosteal flap is reflected to cover the proximal part of the graft entering the femoral tunnel with the cambium layer facing towards the tunnel walls and secured using vicryl no. 2-0; afterward, the femoral end of the graft is fixed to a fixed-loop EndoButton

The grafts were tripled or quadrupled according to their length and diameter; as a result, the final graft was ≥ 8 mm in diameter and ≥ 8 cm in length. The periosteal layer was reflected to cover the proximal 1.5–3 cm of the graft entering the femoral tunnel, with the cambium layer directed outwards to face the femoral bony tunnel and sutured with vicryl 2–0. The femoral end of the graft was fixed to a fixed-loop EndoButton (Smith & Nephew, Andover, MA, USA) (Fig. 2).

The femoral tunnel was drilled using an inside-out transportal approach followed by tibial tunnel creation at the center of the ACL footprint. Tibial remnants were preserved as much as possible. The graft was passed intraarticularly from the tibial side, and the button was flipped, secured at the lateral femoral cortex, and checked using an image intensifier. After passage of the graft, the tibial remnant length covering the graft was measured and recorded. The knee was cycled multiple times and the graft was fixed on the tibial side with maximal manual tension at 20° of knee flexion and neutral rotation using a bioabsorbable screw (Smith & Nephew, Andover, MA, USA), with a diameter similar to that of the tibial tunnel. The wounds were closed in a standard fashion, and an intraarticular drain was placed.

### Postoperative rehabilitation

Postoperative rehabilitation protocol was the same for all patients in both groups, the knee was immobilized in a brace in full extension for the first 2 weeks; meanwhile, aided partial weight-bearing was allowed as tolerated. Isometric quadriceps and straight leg-raising exercises were initiated as early as possible to achieve full extension. After 2 weeks, movement within a protected range of motion (ROM) of 0° to 90° was permitted. At 6 weeks, free ROM was allowed with no restrictions. After that period, under the supervision of a physiotherapist, rehabilitation and conditioning program was initiated and patients were allowed to resume sports at 6 months; pre-injury sports activities were limited until 9 months later.

### Methods of evaluation

- a. Subjective scores:

All patients participating in the study completed the International Knee Documentation Committee (IKDC), Lysholm, and Tegner activity scale subjective questionnaires preoperatively and at the final follow-up. The visual analogue scale (VAS) was used to measure pain intensity at the harvest site at 3, 6, and 12 months.

- b. Objective evaluation:

Knee stability was graded and recorded using the Lachman and pivot shift tests preoperatively and at a minimum of 2 years postoperatively. The Lachman test was graded as 0 (negative), 1 (positive with firm endpoint), or 2 (positive with no endpoint), and the pivot shift test as 0 (absent), 1 (glide), 2 (jerk), or 3 (subluxation). Radiologically, the anterior tibial translation (ATT) was measured using a weight-bearing single-leg stance lateral radiograph at 20º of knee flexion at a minimum of 2 years postoperatively. ATT is defined as the distance between two lines parallel to the posterior tibial cortex, the first tangent to the posterior aspect of the medial tibial plateau and the second tangent to the posterior femoral condyles.

- c. MRI evaluation:

Graft ligametization was evaluated using MRI performed 1 year postoperatively. The following parameters were evaluated: signal-to-noise quotient (SNQ) [10, 11], femoral tunnel aperture widening, graft healing to the femoral tunnel walls based on Ge et al. classification [12], and graft maturity according to the Howell scale [13].

Knee MRI was performed after 1 h of rest. A 3-T MRI unit (Magnetom Skyra, with a 15-channel phased-array send/receive knee coil; Siemens AG Healthcare) was utilized; a protocol that allowed multi-planar T2-weighted reconstructions parallel and perpendicular to the graft was used. Additional MR sequences included oblique sagittal 2D T2 sequencing along the intraarticular segment of the graft, T2 mapping, sagittal and axial 2-dimensional (2D) short tau inversion recovery (STIR), and coronal 2D proton density.

**Fig. 3:**
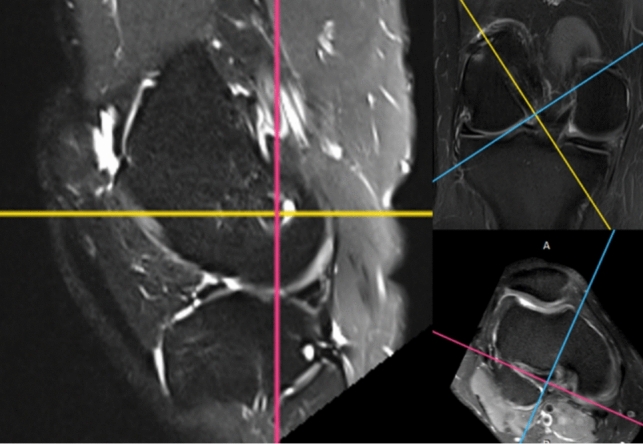
multi-planar reconstruction is used to obtain an image perpendicular to the femoral tunnel aperture where circumferential complete tunnel wall was seen

The MRI images were imported into a DICOM viewer (Radi-Ant DICOM Viewer 2020.2.2; Medixant Company, Poznan, Poland). According to a previously validated measuring technique using multi-planar imaging perpendicular to the entire tunnel [14], femoral aperture widening, graft-tunnel healing, and graft maturation inside the femoral tunnel were analyzed using the first reconstructed image slice placed perpendicular to the tunnel direction where complete circumferential tunnel walls were seen (Fig. 3). The intraarticular graft SNQ was measured using oblique sagittal proton density-weighted fat-suppressed (PD-FS) images.

### Signal-to-noise quotient (SNQ)

The SNQ was used to evaluate the mechanical properties and ligamentization of the graft, as validated in previous studies [11, 15]. The signal intensity was measured on oblique sagittal PD-FS images in 0.05 cm^2^ circular regions of interest (ROI), tangent to the intraarticular ACL cross-section. The graft signal was measured intraarticularly using three equally spaced ROIs at the proximal, middle, and inferior thirds of the graft, and the average value was calculated. The proximal graft ROI was set adjacent to the femoral aperture. The distal graft ROI was set proximal to the tibial aperture, and its distal edge was aligned to the tibial joint surface. The midsubstance graft ROI was set such that its center was midway between the two previously placed ROIs at the level of the anterior and posterior intercondylar notches. The graft signal values were averaged as described by Weiler et al. [11] using a ROI placed at the base of the PCL, central to its broad tibial attachment. The background signal was measured 2 cm anterior to the patellar tendon. The mean signal intensity and standard deviations of each region were recorded based on image pixels as the absolute signal intensity, with a measurement accuracy of one decimal.

Afterward, the SNQ in each of the three graft regions was calculated to standardize the signal intensity using the following formula: SNQ = (graft signal—PCL signal) / background signal.

### Femoral tunnel aperture widening

Femoral tunnel aperture widening was calculated by measuring the difference between the postoperative MR-derived tunnel diameter and the initial tunnel diameter drilled during surgery.

### Graft-tunnel healing

According to Ge et al. [12], graft-tunnel healing was graded based on the signal intensity at the bone-graft interface as follows: (1) Complete healing with low signal intensity and no fluid at the bone-graft interface, (2) Partial healing with high signal intensity detected at a portion of the bone-tunnel interface, and (3) Poor healing with a high signal over the entire circumference of the bone-tunnel interface with poor attachment.

### Graft maturity

Graft maturity in the femoral tunnel was measured using a 4-grade system according to Howell et al. [13] as follows:

(I) Homogeneous, low signal intensity like the PCL and patellar tendon.
(II) Low signal intensity over at least 50% of the graft volume intermingled with portions with increased signal intensity.
(III) High signal intensity over at least 50% of the graft volume intermingled with portions that had a normal ligament signal.
(IV) Diffuse high signal intensity without normal ligament appearance.

The MRI scans were analyzed by two orthopedic surgeons (A.A. and H.H.), and all measurements were calculated twice with a 2-week interval and the mean was utilized. Each rater was blinded to the patient’s group and the score assigned by the other rater. The intraclass correlation coefficient (ICC) with a 95% confidence interval (CI) was calculated to assess interobserver reproducibility. The reliability of the mean (between raters 1 and 2) for the SNQ measurement, femoral aperture widening, graft- tunnel healing, and graft maturity was ICC 0.75 (95% CI 0.61–0.89), 0.70 (95% CI 0.58–0.82), 0.78 (95% CI 0.68–0.88), 0.79 (95% CI 0.71–0.87) respectively.

### Statistical analysis

Data were inputted in the computer and analyzed using IBM SPSS Statistics 20.0 (Armonk, NY: IBM Corp). Categorical data were presented as numbers and percentages. Continuous data were tested for normality using the Shapiro–Wilk test. Quantitative data were expressed as range (minimum and maximum), mean, standard deviation, and median. Student’s *t* test was used to compare two groups for normally distributed quantitative variables, whereas the paired *t *test was used to compare two periods for normally distributed quantitative variables. The Mann–Whitney test was used to compare two groups for non-normally distributed quantitative variables, whereas the Wilcoxon signed-rank test was used to compare the abnormally distributed quantitative variables between two periods. The Friedman test was used to compare abnormally distributed quantitative variables between more than two periods or stages, This was a pilot study, and a priori power analysis was not performed; the Dunn's post-hoc test was used for pairwise comparisons. The significance level of the results was set at 5%.

## Results

### Subjective evaluation

The IKDC and Lysholm scores improved significantly in both groups (Table 2). Moreover, no significant differences in IKDC and Lysholm scores between the groups were detected at the final follow-up. Pre-injury Tegner score was higher in the PA-ACLR group than in the ST-ACLR group; this significant difference was maintained at the final follow up (Table 2). None of the patients had a ROM deficit at 24 months postoperatively. There was a significant difference in the VAS scores between the groups at 3- and 6-months point. Patients in the PA-ACLR group experienced more pain and tenderness at the harvest site early postoperatively. However, pain at the harvest site gradually improved over time, resulting in similar VAS scores between the two groups at 12 months postoperatively (Table 3).

**Table 2:** Comparison between subjective scores of both groups preoperative and at the time of final follow up

		ST-ACLR (*n* = 25)	PA-ACLR (*n* = 23)	*p*
IKDC	Preoperative			
	Mean ± SD	52.3 ± 8.1	50.5 ± 5.9	0.378
	Median (range)	55 (40–69)	50 (41–61)	
	Final follow-up			
	Mean ± SD	85.2 ± 3.8	86.4 ± 3.2	0.247
	Median (range)	86 (77–91)	87 (80–91)	
	^**t1**^**p**_**1**_	< 0.001^*^	< 0.001^*^	
Lysholm	Preoperative			
	Mean ± SD	52.6 ± 4.6	51.7 ± 5.9	0.556
	Median (range)	54 (43–59)	51 (42–60)	
	Final follow-up			
	Mean ± SD	94 ± 3	93.3 ± 3	0.422
	Median (range)	94 (88–98)	93 (88–98)	
	^**t1**^**p**_**1**_	< 0.001^*^	< 0.001^*^	
Tegner	Preoperative			
	Mean ± SD	6.4 ± 0.8	6.9 ± 0.9	0.038^*^
	Median (range)	6 (5–8)	7 (5–8)	
	Final follow-up			
	Mean ± SD	5.7 ± 0.5	6.6 ± 0.8	< 0.001^*^
	Median (range)	6 (5–7)	7 (5–8)	
	^**t1**^**p**_**1**_	< 0.001^*^	0.002^*^	

*t*_*1*_ paired *t *test, *p* p value for comparing between the studied groups, *p*_*1*_ p value for comparing between pre and postoperative scores

^*^Statistically significant at *p* ≤ 0.05

**Table 3:** Comparison between visual analogue scores (VAS) of both groups at 3, 6- and 12-month post-surgery

		ST-ACLR (*n* = 25)	PA-ACLR (*n* = 23)	*p*
VAS	3 months			
	Mean ± SD	0.7 ± 0.8	4.7 ± 0.9	< 0.001^*^
	Median (Min. –Max.)	1 (0–2)	5 (3–6)	
	6 months			
	Mean ± SD	0.3 ± 0.5	3.3 ± 0.6	< 0.001^*^
	Median (Min. –Max.)	0 (0–1)	3 (2–5)	
	12 months			
	Mean ± SD	0.1 ± 0.3	0.5 ± 0.6	0.442
	Median (Min.–Max.)	0 (0–1)	0 (0–1)	

^*^Statistically significant

### Objective evaluation

Lachman test, pivot-shift grading, and ATT improved significantly in both groups. At final follow-up, none of the knees in either group showed significant laxity. There was no significant difference in the aforementioned parameters between the groups at the final follow-up (Table 4).

**Table 4:** Comparison of the lachman test, pivot shift, and anterior tibial translation (ATT) between both groups

		ST-ACLR (*n* = 25)	PA-ACLR (*n* = 23)	*P*
Lachman	Preoperative			
	Mean ± SD	2 ± 0	2 ± 0	1.000
	Final follow up			
	Mean ± SD	0.1 ± 0.3	0.1 ± 0.3	0.711
	Median (range)	0 (0–1)	0 (0–1)	
	^**Z**^**p**_**1**_	< 0.001^*^	< 0.001^*^	
Pivot shift	Preoperative			
	Mean ± SD	1.1 ± 0.6	1.1 ± 0.5	0.979
	Median (range)	1 (0–2)	1 (0–2)	
	Final follow up			
	Mean ± SD	0.4 ± 0.5	0.2 ± 0.4	0.283
	Median (range)	0 (0–1)	0 (0–1)	
	^**Z**^**p**_**1**_	0.001^*^	< 0.001^*^	
ATT	Preoperative			
	Mean ± SD	3.9 ± 0.7	4.2 ± 0.9	0.291
	Median (range)	4 (2.7–5.2)	4.1 (3–5.8)	
	Final follow up			
	Mean ± SD	1.1 ± 0.6	0.9 ± 0.6	0.191
	Median (range)	1.1 (0–2)	0.7 (0–2)	
	^**t1**^**p**_**1**_	< 0.001^*^	< 0.001^*^	

*SD* standard deviation, *t* student’s *t* test, *t*_*1*_ Paired *t* test, *Z* Wilcoxon signed ranks test, *p* *p* value for comparison between the studied groups, *p*_*1*_ *p* value for comparison pre- and post-intervention

^*^Statistically significant at *p* ≤ 0.05

### SNQ values

**Fig. 4:**
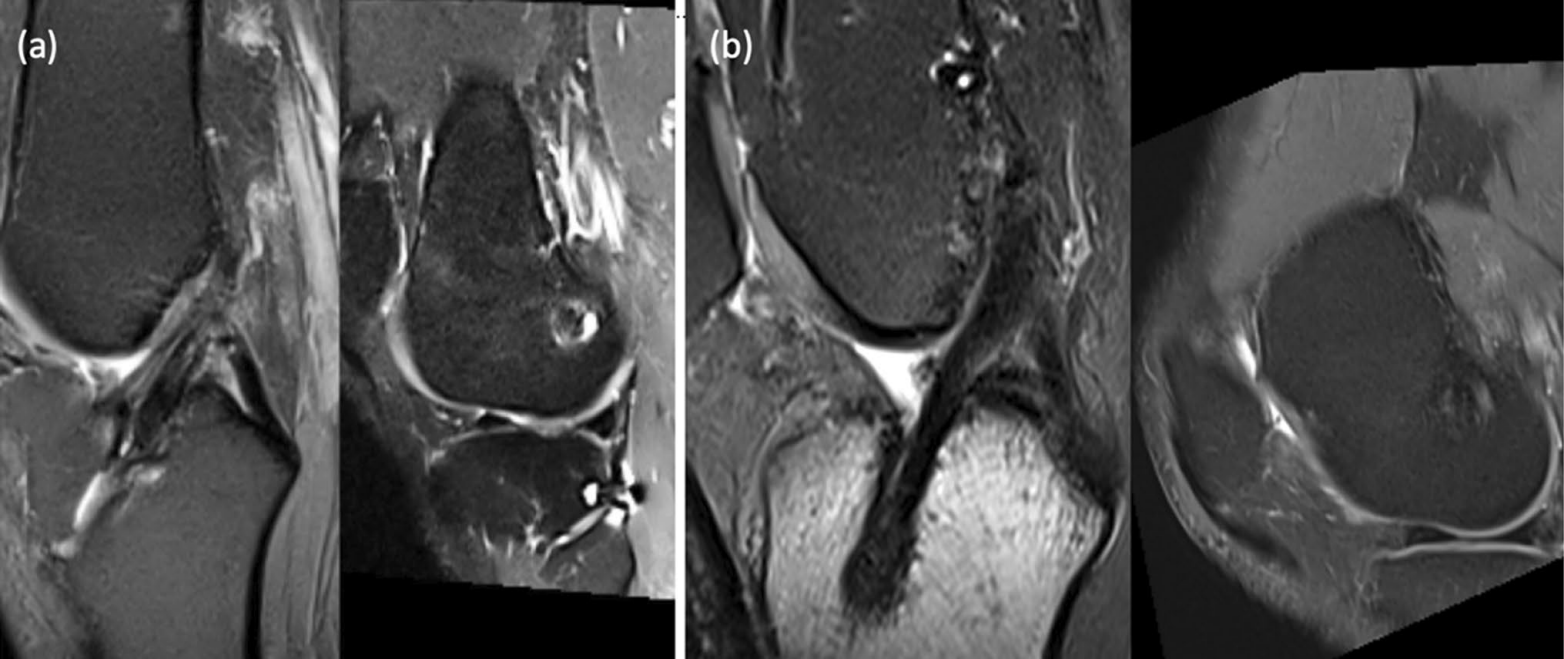
MRI scans obtained 12-month post ACL reconstruction of two matched patients, one from each group. **a** Standard reconstruction with intermediate signal intensity at the proximal part of the graft, poor healing at the femoral aperture (grade 3) and good graft maturity (grade 2). **b** Periosteal augmentation reconstruction with homogenous low signal intensity of the whole graft with good healing at femoral aperture (grade 1) and graft maturity grade 2

The mean SNQ was lower in the PA-ACLR group (3.9 ± 2.2) than in the ST-ACLR group (6.1 ± 4.7), but there was no significant difference (*P* = 0.143). Analysis of the values of the three graft segments (proximal, middle, distal) is presented in Table 5. There was a significant difference between the two groups only at the proximal third of the graft (*P* = 0.005), with the PA-ACLR group showing a consistently low value at the proximal third in most cases (Fig. 4), whereas no differences were identified in the middle or distal portion between both groups (*P* = 0.992, 0.625).

**Table 5:** Comparison between both groups regarding SNQ values

	ST-ACLR (*n* = 25)	PA-ACLR (*n* = 23)	*p*
	Mean ± SD	Mean ± SD	
SNQ proximal	9.6 ± 9.2	2.9 ± 3.3	0.005^*^
SNQ middle	5.5 ± 3.9	5.2 ± 2.8	0.992
SNQ distal	3.3 ± 2.8	3.5 ± 2.7	0.625
SNQ mean^#^	6.13 ± 4.73	3.86 ± 2.17	0.143

*SD* standard deviation, *p* *p* value for comparing between the studied groups

^*^Statistically significant at *p* ≤ 0.05

### Graft healing, graft maturity, and femoral tunnel widening

Significant differences were observed between the two groups when graft healing, graft maturity, and femoral tunnel widening were compared. The PA-ACLR group showed better graft-tunnel healing and graft maturity, with significantly less femoral tunnel widening than the ST-ACLR group (Table 6).

**Table 6:** Comparison between both groups regarding different MRI parameters

	ST-ACLR (*n* = 25)	PA-ACLR (*n* = 23)	*p*
Graft-Tunnel healing	*n* (%)	*n* (%)	
Grade 1	7 (28%)	15 (65%)	0.006^*^
Grade 2	15 (60%)	8 (35%)	
Grade 3	3 (12%)	0 (0%)	
Median (Min.–Max.)	2 (1–3)	1(1–2)	
Graft maturity	*n* (%)	*n* (%)	
Grade 1	4 (16%)	12 (52%)	0.001^*^
Grade 2	9 (36%)	10 (43%)	
Grade 3	9 (36%)	1 (5%)	
Grade 4	3 (12%)	0 (0%)	
Median (Min.–Max.)	2 (1–4)	1 (1–3)	
% of Femoral tunnel widening	30.3 ± 18.3	2.3 ± 9.9	< 0.001^*^

*SD* standard deviation, *t* student *t* test, *p* *p* value for comparing between the studied groups

^*^Statistically significant at *p* ≤ 0.05

### Correlation between different variables

There was no correlation between the final subjective scores (Lysholm and IKDC) at 2 years postoperative and the MRI parameters obtained 1 year after surgery. However, a negative linear correlation was detected between the SNQ of the proximal part of the graft and Tegner activity scale in the PA-ACLR group (*r* = -0.581, *p* = 0.004).

## Discussion

The most important finding of this study was that periosteal augmentation of the hamstring autograft, compared to standard ACL reconstruction, improved graft healing to the femoral tunnel walls, reduced femoral tunnel widening, and was associated with lower SNQ values of the intraarticular proximal part of the graft. These objective findings did not translate into significant clinical differences in patient-reported outcomes 2 years postoperatively. However, we observed a correlation between a lower SNQ score of the proximal part of the graft and a higher Tegner activity scale in the PA-ACLR group.

Graft healing to the bone tunnel has been a major concern that was extensively studied [4, 5, 16].

Experimental studies have demonstrated that periosteal tissue when placed in the graft-bone interface with the cambium layer facing the bony walls can induce bone and cartilage formation resulting in direct tendon to bone healing with a progressive increase in the interface failure strength [17–19]. Clinical studies enforced the use of the periosteum as a biological agent to enhance graft healing [6–8]. Chen et al. [8] reported mid-term results of arthroscopic transtibial single-bundle ACL reconstruction with periosteum-augmented hamstring tendon graft where two separate free periosteal flap were sutured to the hamstring tendon at the contact points with the femoral and tibial aperture. The authors reported excellent clinical scores, with good graft healing and minimal tunnel widening.

In the present study, a modification to the originally described periosteal augmentation technique was adopted, in which the periosteum was harvested, kept attached to the hamstring graft and implanted as one unit for better revascularization and graft incorporation after implantation. The periosteum was reflected so that the cambium layer faced the tunnel walls and covered the whole segment of the graft entering the femoral tunnel to maximize femoral graft integration; the tibial side of the graft was enhanced by preserving the tibial remnants of the native ACL.

There is growing evidence that femoral tunnel graft healing and integration are inferior in comparison to the tibial tunnel [20–24]. Putnis et al. [21] suggested that the femoral-side graft may be the rate-dependent area for healing of the entire graft and concluded that ACL graft rupture after 1 year is associated with MRI appearances of a high graft signal adjacent to and within the femoral tunnel aperture. In the current study, the periosteal flap was used in a way to maximize femoral graft integration and accelerate graft healing to the tunnel walls, thereby decreasing graft motion at the femoral aperture with a subsequent reduction in femoral tunnel widening.

MRI has proven to be a safe, noninvasive measure for evaluating ACL graft remodeling, mechanical properties, and tensile strength [25, 26]. The SNQ is used to assess graft edema and subsequently graft vascularity and biomechanical properties, as it is inversely proportional to the graft’s tensile strength [11, 27]. The mean SNQ of hamstring autograft 1 year after surgery was reported in various studies to be (1–22) [28–30]. Liu et al. [31] reported SNQ values of detached hamstring autograft (18.3 ± 7.7), compared to those of hamstring tendons, remained attached distally (12.6 ± 7.0). Cavaignac et al. [28] reported SNQ values of (5.9 ± 3.7) for quadrupled semitendinosus graft and (5.2 ± 4.5) for doubled semitendinosus and gracilis grafts. The mean SNQ in this study was relatively low in both study groups (ST-ACLR, 6.1 ± 4.7; PA-ACLR, 3.9 ± 2.8), which may be due to patient selection criteria, as we tried to control for variables that could affect graft healing such as meniscal deficiency, elevated PTS, high grade pivot, small graft size, and absence of tibial remnants [32–34], as well as adopting a slow rehabilitation program [35].

Higher SNQ values in the proximal part of the ACL graft (range, 12–23.2) compared to the intraarticular and tibial portions are consistent findings reported in MRI-based graft healing studies [31, 36]. In our study, SNQ of the proximal portion of the graft was consistently lower in the PA-R group (2.9 ± 3.3) than in the ST-R group (9.6 ± 9.2) and even lower than values reported in the literature [31, 37] this may lead to probably better graft rupture rate based on findings in previous studies [21]. The SNQ values of the middle and distal portions of the graft were consistently low in both study groups, which may be attributable to the preservation of the tibial remnant of the native ACL, as these values are consistent with those reported in another study investigating the effect of tibial remnant preservation [38].

In the present study, the tunnel aperture was selected for analysis to evaluate tunnel widening and graft-tunnel healing because of existing histological evidence that it is an important site for graft incorporation [16, 24]. Previous studies have demonstrated inferior graft healing to the femoral tunnel walls in comparison to the tibial tunnel, with complete healing ranging from (21–26%) [39, 40]. In the present study, graft-tunnel healing at the aperture was considered complete (grade 1) in 65% of patients in the PA-ACLR group compared to 28% of patients in the ST-ACLR group, which contributes to existing evidence that the periosteal flap could enhance the graft-bone interface and promote healing through direct and indirect integration, as discussed previously.

Significant tunnel widening with the use of hamstring tendon autografts has been previously reported [24, 41]. In the present study, significant reduction in femoral tunnel widening percentage could be detected in the PA-ACLR group (2.3% ± 9.9%) compared to the ST-ACLR group (30.3% ± 18.3%). Moreover, tunnel narrowing was detected in few cases in the PA-ACLR group (24%), and this may be attributed to the osteogenic properties of the periosteal tissue, these values are comparable to those of the bone patellar tendon bone (BTB) graft [42, 43]. Robert and Es-sayeh [44] studied the effect of the periosteum in preventing femoral tunnel widening and found that there was a significant reduction in femoral tunnel enlargement at the tunnel outlet with the use of a periosteal flap compared to standard reconstruction, although tunnel widening was constant in both study groups.

The clinical significance of the MRI-based graft appearance, tunnel widening, and graft tunnel integration remains unclear [45, 46]. In the current study, there was no significant correlation between the MRI parameters studied and clinical scores (IKDC, Lysholm) at 2 years postoperative, with both groups having good-to-excellent IKDC and Lysholm mean scores, as well as nearly normal ATT, indicating that patients in both cohorts had little to no limitation with daily or sporting activities and minimal or zero symptoms postoperatively. The VAS scores at 3- and 6-month postoperatively differed significantly between both groups. Patients in the PA-ACLR group experienced more pain at the harvest site, which was expected due to periosteum stripping at the anteromedial tibial border; however, the patients experienced gradual improvements. At 2 years post-operatively, both groups experienced no pain.

There was a weak negative linear correlation between the proximal intraarticular graft signal and Tegner scale, with the correlation reaching significance only in the PA-ACLR group (moderate correlation, *r* = −0.581, *p* = 0.004). The median postoperative Tegner score in the ST-ACLR group was 6 (pre-injury score: 6, range: 5–8) and 7 (pre-injury score: 7, range: 5–8) in the PA-ACLR group, indicating that patients in both groups returned to near their pre-injury levels of activity. Despite having a higher pre-injury Tegner activity score, a large percentage of patients in the PA-ACLR group returned to their high pre-injury level with no failures at 2-year follow up; these findings are consistent with those of previous studies correlating the high SNQ of the femoral portion of the graft with inferior outcomes [21, 30, 46].

This study had several limitations. First, due to the lack of relevant data, we were not able to calculate the appropriate sample size in advance and the small number of participants might have resulted in a relatively low statistical power. Second, graft SNQ is influenced by several variables, including knee position at the time of the scan and technical issues such as sequence and scanner characteristics, reconstruction algorithms, gray scale displays, surgical technique, and graft bending angle [30]. These differences raise doubts about the use of MRI signal intensity measurements as a quantitative method for measuring graft maturation. Third, the heterogeneity of methods for measuring signal intensities and MRI slice selection for measuring tunnel widening and graft healing prevented a direct comparison between studies of similar interest. Four, MRI was performed at least 1 year after surgery in all patients, although studies have shown that graft maturation and normalization of MRI signals can progress up to 2 years postoperatively [47]. However, despite the continuation of ligamentization, it is important to evaluate the SNQ at 1-year post-surgery, a time point at which most patients want to return to sports. Fifth, anterior tibial translation measured on a standing single leg stance lateral radiograph is greatly influenced by the PTS and it may not be suitable for patients’ comparison, however, this influence is minimized since we included only patients with PTS less than 12 degrees.

## Conclusion

Periosteal wrapping of hamstring tendon autografts is associated with improved graft-tunnel healing and maturation, as well as a lower incidence of femoral tunnel widening based on MRI analysis 1-year after ACL reconstruction. However, the patient-reported outcomes and measured laxity were similar between the two study groups at 2 years postoperative evaluation.

## Data Availability

Data are available on request to authors.

## Abbreviations

ACL: Anterior cruciate ligament
PCL: Posterior cruciate ligament
LCL: Lateral collateral ligament
ST-ACLR: Standard anterior cruciate ligament reconstruction
PA-ACLR: Periosteal augmented anterior cruciate ligament reconstruction
ATT: Anterior tibial translation
ROI: Region of interest
IKDC: International knee documentation score
SNQ: Signal to noise quotient
MRI: Magnetic resonance imaging
VAS: Visual analogue scale
BTB: Bone patellar tendon bone graft
